# Microbial Groundwater Quality Status of Hand-Dug Wells and Boreholes in the Dodowa Area of Ghana

**DOI:** 10.3390/ijerph15040730

**Published:** 2018-04-12

**Authors:** George Lutterodt, Jack van de Vossenberg, Yvonne Hoiting, Alimamy K. Kamara, Sampson Oduro-Kwarteng, Jan Willem A. Foppen

**Affiliations:** 1 Department of Civil Engineering, Central University, Miotso-Campus, Miotso Tema, Ghana; glutterodt@central.edu.gh; 2 Department of Environmental Engineering and Water Technology, IHE Delft Institute for Water Education, Westvest 7, 2611 AX Delft, The Netherlands; kolif.02@gmail.com; 3 Department of Water Science and Engineering, IHE Delft Institute for Water Education, Westvest 7, 2611 AX Delft, The Netherlands; y.hoiting01@gmail.com (Y.H.); j.foppen@un-ihe.org (J.W.A.F.); 4 Department of Civil Engineering, Kwame Nkrumah University of Science and Technology (KNUST), Kumasi, Ghana; sokwarteng@gmail.com

**Keywords:** groundwater quality, Adenovirus, Rotavirus, *Escherichia coli*

## Abstract

To assess the suitability of water sources for drinking purposes, samples were taken from groundwater sources (boreholes and hand-dug wells) used for drinking water in the Dodowa area of Ghana. The samples were analyzed for the presence of fecal indicator bacteria (*Escherichia coli*) and viruses (Adenovirus and Rotavirus), using membrane filtration with plating and glass wool filtration with quantitative polymerase chain reaction (PCR), respectively. In addition, sanitary inspection of surroundings of the sources was conducted to identify their vulnerability to pollution. The presence of viruses was also assessed in water samples from the Dodowa River. More than 70% of the hand-dug wells were sited within 10 m of nearby sources of contamination. All sources contained *E. coli* bacteria, and their numbers in samples of water between dug wells and boreholes showed no significant difference (*p* = 0.48). Quantitative PCR results for Adenovirus indicated 27% and 55% were positive for the boreholes and hand-dug wells, respectively. Samples from all boreholes tested negative for the presence of Rotavirus while 27% of the dug wells were positive for Rotavirus. PCR tests of 20% of groundwater samples were inhibited. Based on these results we concluded that there is systemic microbial and fecal contamination of groundwater in the area. On-site sanitation facilities, e.g., pit latrines and unlined wastewater drains, are likely the most common sources of fecal contamination of groundwater in the area. Water abstracted from groundwater sources needs to be treated before use for consumption purposes. In addition, efforts should be made to delineate protected areas around groundwater abstraction points to minimize contamination from point sources of pollution.

## 1. Introduction

Groundwater from shallow wells, boreholes and springs remains a major source of water for various uses in Sub-Saharan Africa [1,2] and other parts of the world. Tapping groundwater from shallow aquifers comes with water quality challenges, especially when these wells are provided near beneficiary communities and within homes. Even more protected deep boreholes [3] are also sometimes polluted by on-site sanitation facilities when there is a hydrologic connection between deep aquifers and younger geologic layers on the surface where all on-site sanitation systems are sited. Such a situation is more evident in low income communities and peri-urban areas in Sub-Saharan Africa, with onsite sanitation systems predominantly used in the areas [4]. The presence of on-site sanitation systems renders groundwater sources vulnerable to pollution by microbial pathogens.

Evidence from previous studies in different countries within the Sub-Saharan Africa region shows that groundwater sources; boreholes [1,5,6], springs [2,7] and hand-dug wells [8] may be polluted with microbial pathogens originating from the intestinal tract of humans and warm blooded animals. The link of improper on-site sanitation facilities in poor urban and rural areas to groundwater pollution is common in the literature [4,9,10,11,12]. Microbial pathogens from human excreta from pit latrines and effluents from on-site sanitation facilities may therefore lead to disease outbreaks [13]. Therefore, monitoring and assessment of the microbial quality of water from boreholes and hand-dug wells is important, as findings will inform effective management strategies to improve the quality of water from these sources. 

Dodowa is a peri-urban poor community of the Shai-Osu Doku district of Ghana, and groundwater serves as an important source of water for various uses including drinking [14]. Despite additional supplies from treated surface water, the community continue to extract groundwater from wells and boreholes for their various water needs as a substitute/alternative to treated piped water. This is evidenced by the more than 80 hand-dug wells and boreholes currently in use within the community. The dependence on groundwater is largely attributable to the unreliable supply of water from the Water Company and in some cases the inability of the poor in the community to afford the cost of treated water. 

Like many other communities in Ghana the township has no sewage treatment facilities, and therefore relies on on-site sanitation for the disposal of excreta, wastewater, and garbage from homes. The pit latrines, unlined drains, waste dumps and faulty septic tanks in the Dodowa area are known to be common sources of microbial contamination of groundwater and chemical contaminants, e.g., nitrates [9]. These sources are likely to pollute groundwater sources in the area, especially the shallow hand-dug wells with depths ranging from l.5 to 10 m. Gibson et al. [15] have reported the lack of monitoring and evaluation strategies of drinking water quality in Ghana and other developing countries, which is also true for Dodowa. The continuous use of untreated groundwater in the area may therefore pose a public health threat to the community. 

In this work, risk factors to contamination of selected boreholes and hand-dug wells in the Dodowa area are surveyed, the suitability of water for drinking from these groundwater sources is assessed by analyzing for fecal indicator *E. coli* bacteria that are commonly used as primary/traditional bacterial indicator in drinking water quality assessments. In addition, viruses in samples of water from the wells and boreholes are analyzed, and the safety and suitability of water from these sources for drinking are assessed. 

## 2. Materials and Methods

### 2.1. The Study Area

Dodowa is a peri-urban poor community between latitude 5.87 and 5.91 °N, and longitude 0.06 and 0.12 °W in the Greater Accra Region of Ghana, West Africa, and the capital of the Shai Osudoku District. The area is drained by the Dodowa river which takes its source from hills (Akwapim-Togo ranges) and traverses through the central part of the township to the south and beyond Dodowa where it flows into the Volta river. Two rainfall peaks occur in the area, the major rainy season occurs from May to July and the minor occurs between September and October, with mean rainfall of 900 mm/year. The average temperature in the area is 27 °C [16]. According to the 2010 census report by the Ghana Statistical Service, some 12,075 people live in the Dodowa Community [17]. In Dodowa water is accessed through a number of sources, including supplies from the Ghana Water Company Limited, and more than one-third of the population rely on self-supplies from groundwater sources (hand-dug wells and boreholes) [14]. 

### 2.2. Sanitary Risk Inspection 

A sanitary risk survey involving the identification of potential point sources of microbial contamination was performed for 46 hand-dug wells and 12 boreholes in the area to identify their risk level to microbial contamination. The sanitary inspection method was adapted from the British Geological Survey and has previously been used by Howard et al. [2], it is based on a checklist for 11 contamination risk factors to groundwater from on-site sanitation. The procedure involved physical inspection of the sources followed by inspection of the surrounding environment and recording their status (Yes or No) on the 11 commonly identified risk factors (Table 1). Parameters were level of protection, proximity to septic tank and pit latrines uphill or downhill. A yes answer indicated the presence of a risk factor of microbial contamination around the water source. A No answer indicating the absence of risk of microbial contamination. A final risk score was obtained by totaling the score for each source to obtain a risk score on a scale of 1–11. The total scores were further grouped into very high for a total score of 9–11, high for 6–8 and score ranges of 3–5 and 0–2 were assigned intermediate and low risk, respectively. 

### 2.3. Bacteriological Quality Assessment 

Samples of water from 11 hand-dug wells and 11 boreholes were taken for bacteriological quality assessment. To do this, 250 mL of water from a source (borehole or hand-dug well) water was collected in sterile polypropylene bottles and by means of a syringe 100 mL of each sample was passed through a 0.45 µm cellulose acetate filter, the filter was then placed on Chromocult agar (MerckMillipore, Darmstadt, Germany) plate and incubated at 37 °C for at least 18 h. This was followed by counting of *Escherichia coli* colonies on the agar plates. 

### 2.4. Bacteria Growth Experiments

To assess the possibility of bacteria re-growth in the wells, single colonies of *E. coli* cells were isolated from Chromocult agar plates with a sterile tooth pick and incubated in nutrient broth (Hi Media Laboratories, Vadhani, India) for 24 h at room temperature. The cell solution was then centrifuged, and the pellets washed with groundwater filtered through 0.45 µm cellulose acetate paper and re-suspended in 200 mL of pre-filtered groundwater in sterile 250 mL plastic bottles. The 250 mL plastic bottles were then kept at room temperature for a period of 7 days over which samples were taken and plated on Chromocult agar to assess the changes in cell suspension with time.

### 2.5. Viruses Concentration 

Virus concentration experiments were conducted for groundwater samples from 11 selected hand-dug wells and from 11 boreholes at different depths. In addition, ten samples were collected from the Dodowa stream.

For the experiments 100 L and 10 L of water samples from groundwater sources (boreholes and wells) and surface water, respectively, were concentrated. A standard glass wool filtration method previously used by Katukiza et al. [18] was employed. The conditioned water sample was flushed through a 36 g of oiled-white glass-wool (Insulsafe 12, Asbipro, Schiedam, The Netherlands), previously packed to a density of about 0.5 g/cm^3^ in a transparent plastic column of diameter 40 mm. The glass-wool was eluted with beef extract in glycine buffer solution, pH 9.5, and the eluate was then flocculated by lowering the pH with HCl to the pH of maximum flocculation or turbidity, which was 2.8–2.9, for the batch of beef extract used.

The flocculated-eluted sample was centrifuged for 30 min at 2885× *g* in an ALC PK 120 (Cologno Monzese (MI), Italy) centrifuge, equipped with an O-E24 swing-out rotor. Pellets were re-suspended in 10 mL Phosphate Buffered Saline (PBS), pH 7.0. The concentrated samples were aliquoted over two scintillation vials and stored at −20 °C. The samples were transported frozen to the IHE Delft laboratory in Delft, The Netherlands and kept frozen until use.

To test temporal variation in the virus samples we took samples from DW6 and DW7 over a period of 21 days. To assess different cycle time, samples were taken each week, 4 samples were taken within 24 h at day 7 and 8, and daily samples were taken over a week between day 7 and 14. Because of logistic reasons the series for DW7 started 2 days later than the series for DW6.

### 2.6. Nucleic Acids Isolation 

Nucleic Acids (DNA and RNA) were isolated from the concentrated samples, using a guanidine thiocyanate (GuSCN) protocol [19]. To do this 100 µL of concentrated sample was treated by 500 µL GuSCN Buffer and 10 µL silica colloids. After centrifuging, the silica pellets were washed twice with 500 µL wash buffer, twice with 500 µL ethanol and once with 500 µL acetone. After drying the pellet at 56 °C, the nucleic acids were eluted in 55 µL TE Buffer. The mixture was vortexed for 3 min and incubated for 10 min at 56 °C. After centrifuging for 2 min at 13,000× *g*, 50 µL of supernatant was pipetted into a clean vessel. 

For RNA a reverse transcriptase step was performed immediately after the nucleic acids isolation to transcribe the RNA into cDNA. 5 µL of sample was mixed with 0.3 µL random hexamers and 8.7 µL DEPC water, and inserted in a MJ mini thermal cycler (BioRad) for 5 min at 70 °C. Then a mix of 0.3 µL reverse transcriptase, 1 µL (10 mM stock) dNTP’s, 5 µL 5x RT buffer and 4.7 µL DEPC treated water were added. The reaction tubes were then placed into the thermal cycler and held at 25 °C for 10 min, followed by 42 °C for 60 min and 70 °C for 10 min. The DNA and cDNA samples were stored at −80 °C and used for further experiments.

### 2.7. Q-PCR and Nested PCR

A quantitative Polymerase Chain Reaction (PCR) protocol was used to amplify the DNA fragments of Rotavirus and Adenovirus. Each run had a positive control, and a negative control (‘No Template Control’ (NTC)) containing DEPC treated water without DNA or RNA. The positive controls were obtained from the Dutch National Health Institute (RIVM). The efficiency of the PCR procedure was tested using dilution series of a positive control. Efficiencies were calculated using: Efficiency = (10^(−1/slope)^ − 1) × 100. 

The Heim et al. [20] qPCR protocol was used for Adenovirus. Some samples were analyzed using a nested PCR protocol [21]. PCR steps were carried out in 25 µL mixtures of which 4 µL sample (DNA) and 21 µL PCR mix. The PCR mix contained 10x PCR buffer (Sigma-Aldrich), 10 mM dNTP mix (GenScript), 5 U Taq Polymerase (GenScript), DEPC treated water (Sigma Aldrich) and a forward and reverse primer synthesized by Biolegio (Nijmegen, Netherlands) each with an end concentration of 10 µM. 

For the detection of Rotavirus in the samples, a quantitative PCR protocol following the method of Freeman et al. [22] was used on cDNA generated after the nucleic acid isolation step. A qPCR mix with a total volume of 25 µL was prepared from 4 µL cDNA sample and 21 µL mix. The total mix was inserted in the MJ mini thermal cycler (BioRad) for 5 min at 95 °C to activate the Taq Polymerase, then 50 cycles of 20 s at 94 °C and 60 s at 60 °C.

Both for Rotavirus and Adenovirus, every sample was tested at least 3 times. Inhibition of PCR was tested in negative samples by spiking the sample with known amounts of Adenovirus DNA or Rotavirus cDNA.

## 3. Results 

### 3.1. Direct Sanitary Risk Inspection and Bacteriological Quality Assessment

**Figure 1:**
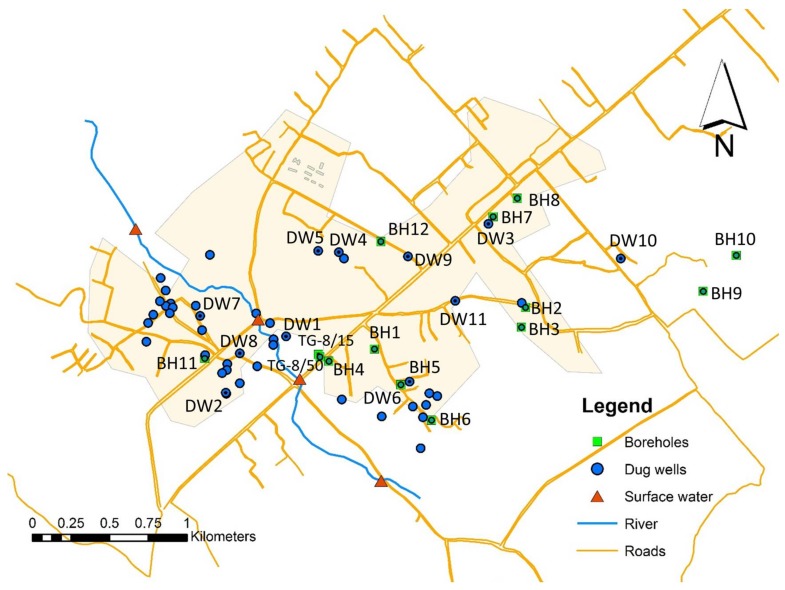
Map of Dodowa with sampling points. The labelled sampling points (black dots) are listed in Table 2.

Results of assessment of groundwater sources in the area are summarized for key contamination risk factors for 12 boreholes and 46 hand-dug wells (Figure 1, Table 1). Four and six important risk factors were respectively, identified for boreholes and hand-dug wells. Results indicate that the quality of water in hand-dug wells in the area are more likely to be affected by on-site sanitation facilities than those of the boreholes. The general observation is that there are no delineated protection zones around the boreholes and the hand-dug wells. Worn-out seals of borehole pumps and the presence of improper on-site sanitation facilities (e.g., sullage drains, animal excreta and waste dumps) within 10 m are the two leading key contamination risk factors observed for boreholes. Site inspection showed that 50% of the boreholes are at risk to each of this two contamination risk factors. Three (25%) of the boreholes are located close to unlined pit latrines with effluents percolating through the soils and one borehole was within 15 to 20 m of an uncapped well which may itself serve as an entry point of pollutants into the surrounding aquifer. Two test boreholes TG-8/50 and TG-8/15 drilled under this research project have been sited in low risk environments.

Substantial numbers (63%) of the hand-dug wells do not have proper top-covering protection in addition to short (<1 m) apron walls; also, results show that for 65% of the wells, latrines, and septic tank soakaways are sited on the upstream side of the wells and within distances of between 10 to 30 m. More than 70% of the hand-dug wells are sited within 10 m of nearby sources of contamination e.g., wastewater drain, waste dumps dump and animal droppings. Other important risk factors to contamination observed within the vicinity of hand-dug wells are the presence of pit latrines and or septic tanks within 10 m of the wells and 48% of the wells with their ropes tied to buckets used for drawing are normally left at points around the wells that are likely to be contaminated by fecal matter. We conclude that hand-dug wells in the area are at greater risk to contamination compared to the boreholes.

### 3.2. Bacteriological Quality of Hand-Dug Wells and Boreholes

Results from groundwater samples from all wells and boreholes indicated fecal contamination with indicator organism *E. coli* present in samples from these wells. From this result we can conclude that there is systematic pollution of groundwater in the study area.

### 3.3. Bacterial Survival Experiments

**Figure 2:**
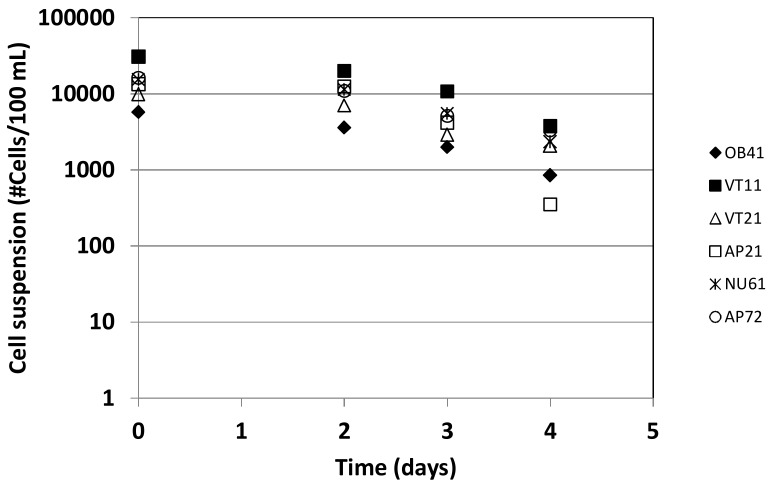
Results of bacteria survival experiments.

Due to the numerous sources of nutrients surrounding the wells we tested if possible leaching of nutrients into the groundwater system in the neighborhood of the wells would favor bacterial regrowth. Such capability would likely influence results of bacteriological assessment. The test results show however decay of cell numbers with time (Figure 2), indicating that conditions in the wells are not favorable for bacterial regrowth and hence the bacteria found in the samples can be attributed to recent supply from fecal matter.

### 3.4. Virus Content in Groundwater

Adenovirus and Rotavirus were found in only a few samples. Apart from the absence of template, negative PCR tests can be attributed to two factors. First is the possibility of a concentration of template (Extracted DNA/RNA) below the detection limit, and the second is the possible presence of inhibitory substances that prevent the amplification of the targeted DNA. To reduce the chance of a negative result due to low template concentrations, at least 3 confirmatory tests were conducted on all samples. Samples were considered positive for the presence of viruses if at least two of the three tests showed positive results. For Rotavirus 13 of all 62 samples were ambiguous and for the Adenovirus samples 48% were ambiguous. Inhibition was tested by spiking samples with target (c)DNA before the PCR. The inhibition test conducted for all negative samples indicated that 19% (12/62) of all samples were inhibited. Table 2 shows qualitative results for virus analyses and contaminant risk score obtained for each groundwater point sampled. Test for the presence of Adenovirus showed positive results for three (27%) of the boreholes (BH1, BH6 and BH11) and six (55%) hand-dug wells (DW1, DW2, DW6, DW7, DW8 and DW10). Samples from all boreholes considered for PCR tested negative for the presence of Rotavirus, but three hand-dug wells (DW4, DW5 and DW7) (27%) tested positive for the presence of Rotavirus. Also, all four samples taken from the Dodowa River tested positive for both Adenovirus and Rotavirus.

Results of PCR inhibition test conducted on 62 samples that tested negative for the presence of viruses indicated that 19% of the samples were inhibited.

Temporal variation was only tested for viruses in DW6 and DW7 over a period of four weeks. No clear trend was found in virus detection. In DW6, 4 samples of Rotavirus were inhibited around day 7, the other samples were negative (i.e., no virus was detected). For DW7, 5 out of 12 samples were ambiguous, the remaining ones were negative. For Adenovirus DW6: 9 out of 12 were doubtful, the rest were negative, DW7: 4 out of 12, around day 7, were positive, 5 were doubtful, one was negative. Although around day 7 the samples had stronger indication of the presence of virus, these data also do not show a clear trend.

## 4. Discussion

### 4.1. Risk Assessment and Bacteriological Quality of Boreholes and Hand-Dug Wells

Hand-dug wells have variously been implicated with microbial contamination of groundwater sources [7]. Results of contamination risk assessment indicated that the shallow hand-dug wells in the area have more sanitary issues that can cause pollution compared to the boreholes (Table 1). This observation is attributable to the siting of the groundwater source types. Most of the boreholes in the Dodowa area have been provided by governmental organizations that apply standards in the siting of boreholes to reduce possible adverse impacts on the quality of water as compared to the construction of hand-dug wells. We have no information about the of the borehole depths that are not indicated in Table 2. The results of this study do however show that the construction, depth and/or locations of these boreholes are not sufficient to prevent against contamination.

Based on the WHO [23] requirement of an absence of *E. coli* cells in 100 mL of drinking water, we conclude that water from all groundwater sources sampled in this study is not suitable for human consumption, unless treated. Despite the theoretically lower vulnerability of boreholes to microbial contamination, all wells and boreholes under this study were contaminated with high counts of *E. coli* bacteria (Table 2). Even the supposed-to-be-sealed boreholes contained high numbers of *E. coli* bacteria, indicating systemic fecal contamination. In addition, the number of cells (*E. coli* and other bacteria) per 100 mL of water samples from boreholes that were assessed with low risk to microbial contamination (TG-8/50 and TG-8/15) were also high. Their *E. coli* numbers were even higher or equivalent to the numbers from high risk boreholes and hand-dug wells, which indicates systemic pollution of groundwater in the area. The observed non-significant difference between *E. coli* numbers in water samples from hand-dug wells and boreholes can be attributed to widespread groundwater pollution within the Dodowa area.

We attribute the high bacterial suspension to a widespread presence of point sources of pollution within the environment. These leach effluent through soils into the groundwater system, defective boreholes, and uncovered wells, in addition to other identified key risk to contamination factors (Table 1). 

Sanitary risk inspection was chosen as primary assessment of possible contaminated sites. Sanitary risk score and microbial contamination in water samples did not show association between the two, for both boreholes and hand-dug wells. Reports of inability of sanitary risk scores to infer microbial contamination of boreholes and hand-dug wells are common in the literature [24,25,26]. In these studies, the non-correlations were due to low bacteria suspension in groundwater sources with high risk to microbial contamination. In our case, the high suspension of bacterial cells in samples from wells can be ascribed to the continuous patronage of pit latrines that are scattered over the township, combined with continuous abstraction of water from the sources. Continuous use of pit latrines and other improper sanitary practices results in a dense concentration of point sources with continuous inoculation of bacteria and other microbial pathogens into the aquifers of the area. These pathogens can spread within the groundwater system through advective transport by flowing groundwater. Abstraction of groundwater for various uses will induce forced convection and facilitate inflow of contaminants from nearby contamination sources. This explanation is supported by the high counts of *E. coli* bacteria in water samples, suggesting recent pollution of groundwater by fecal matter. On top of that, most of the wells sampled are within distances of less than 30 m from potential sources of groundwater contamination (Table 1). There is therefore a possibility of constant inflow of leachate of effluents from the bottom of pit latrines into the nearby wells. High bacteria content in the wells can also be ascribed to two field observations. First the depths of the wells sampled for this work ranged between 1.5 m to less than 7 m from the surface. Second, field observations show that for some of the wells in the area the depth to the water level rises above the surface after the rainy season. These two observations indicate that microbial pathogens from onsite sanitation will have short travel paths to enter into the groundwater system of the area and pollute wells and boreholes. 

For the lower-risk boreholes it is difficult to determine the source of contamination as there are no identified sources of contamination near the boreholes. The possible presence of dual-porosity hydrogeological characteristics in the area may be one of the reasons for the high bacteriological content in boreholes [27]. In dual-porosity hydrogeological systems preferential flow paths ensure rapid groundwater flow in inter-connected geological discontinuities (e.g., fractures, faults and joints) [28,29]. Cells suspended in samples of water from the boreholes may be among a fraction of a low attaching bacterial sub-population that might have been transported over large distances [30,31,32,33]. In our work, bacterial re-growth results indicate that conditions within the saturated zone do not favor bacterial growth. Therefore, we rule out bacteria growth or survival as reasons for high concentrations of bacterial cells within samples. We attribute the contamination of the low risk deep boreholes to a possible combination of preferential flow paths in the aquifer of the area, of low attaching bacterial sub-populations, of low ability of aquifer media to filter bacteriological pathogens from flowing groundwater beneath much of the Dodowa area and of the possibility of detachment of cells from surfaces of the aquifer media.

### 4.2. Presence of Viruses in Water Samples 

Environmental samples usually have very low virus concentrations, due to the inability for viruses to replicate without a host cell and because of dilution effects [34]. Likely because of the low virus concentration, the results turned out to be difficult to reproduce. All samples were tested at least 3 times, but for Rotavirus 21% of all samples were ambiguous and for Adenovirus 48% were ambiguous. Such ambiguous results have previously been described as “Monte Carlo effect” [21]. The Monte Carlo effect depends on the concentration of the template in the sample. The lower the template concentration, the less likely the presence of the template will be reflected in the results. This may make results in positive samples stochastic.

For virus concentration we used volumes up to 100 L. Given the difficult-to-interpret results from the PCR tests this volume seems to enable detection of virus numbers that are just above the detection limit. However, 100 L was the maximum volume that was practical for processing. With 100 L, the samples, especially some of the dug well samples, already polluted and clogged the glass wool filter. Moreover, the total concentration run took several hours. Longer processing would, combined with the elevated temperatures in the provisional lab (30–35 °C), deteriorate the quality of the sample. Therefore, it will be difficult to concentrate larger volumes with the glass wool method.

The results show that generally, bacterial contamination was high in the community. Adenovirus however was positive in many (9/22) of the wells tested than Rotavirus (which was positive in just 1 out of 22).

It was seen from the bacteriological test that dug wells were heavily contaminated with *E. coli*. However, for Rotavirus, only one out of the 62 samples was genuinely positive. The reason could be that there was very low Rotavirus contamination of the environment, or absence of it in most sources of fecal contamination at the time of inspection. There was one ambiguous possible contamination with Rotavirus in the borehole samples, while the dug wells had a few samples that gave an ambiguous or positive signal (Table 2). A general reason could be that dug wells are more liable to viral contamination than boreholes mainly because of their closeness to the surface where there could be higher concentration of fecal contamination and shorter decay time. Rotavirus was not detected in any of the 18 samples from the 11 boreholes tested. The virus could either be absent in the borehole environment, or below the detection limit in the 100 L sample size. Physical and chemical factors such as temperature or chemical composition may also enhance virus removal at deep subsurface level. 

For viral contamination, the prediction of the sanitary inspection was fairly accurate for the dug wells and less accurate for the bore holes as the result on Table 1 and Table 2 indicate. Unlike Rotavirus, some boreholes were positive for Adenovirus. A remarkable outcome of the result of Adenovirus in boreholes was that two of the three positive boreholes were not at high or very high risk of contamination. This points to the fact that, in addition to immediate environmental sanitation, deep groundwater contamination also depends on other factors such as the properties of the contaminant, the hydrogeology, and geochemical characteristics of the area. This also indicated that the risk scores do not scale and that therefore these cannot be used for quantitative assessment of risk. 

## 5. Conclusions

Field work and laboratory experiments were conducted to identify contamination of selected boreholes and hand-dug wells. The vulnerability of the two groundwater source types to microbial contamination was compared. The microbial water quality was tested for viruses (Adenovirus and Rotavirus) and *E. coli* bacteria. From results we conclude:

- There is widespread fecal pollution of groundwater in the area; both hand-dug wells and boreholes are heavily loaded with fecal matter. This situation will not change unless the contaminating risk factors as we found in Table 1 are fixed and sanitary measures are taken.
- Hand-dug wells in the area seem at even greater risk to contamination compared to the boreholes; identified dominant risk factors to contamination are on-site sanitation facilities e.g., pit latrines and unlined pit latrines. However, even the boreholes are not sufficiently constructed or sited to prevent contamination.
- Systemic contamination by combined point sources is the main source of fecal contamination of groundwater sources.
- Based on the WHO [23] drinking water standards, groundwater from all points sampled is not suitable for drinking and needs to be treated before consumption.

### Recommendations

This study shows that the microbiological contamination is high for drinking water that comes from groundwater sources under peri-urban poor settlements. Groundwater remains an important source of water for the people of this community, but measures need to be taken urgently to avoid consumption of such untreated water. Water from groundwater sources need to be disinfected or boiled before use for drinking purposes. It is important for the local governing body, the District Assembly, to come out with regulations for siting of wells and boreholes to ensure adequate distances between the sources and potential point sources of pollution. A law is needed that will enforce the provision of protection zones around facilities used in abstracting groundwater for domestic use in the area. On top of that it is important to do regular repair of broken wells and boreholes. Open wells and faulty borehole caps need to be covered and repaired respectively. Most importantly the community needs to be educated both on activities around the drinking water sources that impair the microbial quality of groundwater and on safe handling of water obtained from these sources.

## Figures and Tables

**Table 1:** Contamination risk factors of boreholes (total 12) and dug wells (total 46) by contamination factors.

	Risk Factors to Boreholes	No. of Boreholes	% of Total Number
Boreholes	Unsanitary/worn-out seal of borehole pump	6	50
	Nearest latrine or a pit latrine that percolates to soil, i.e., not sewered	3	25
	Uncapped well within 15–20 m of the borehole	1	8
	Other environmental source of pollution (e.g., animal excreta, rubbish, and surface water discharge) within 10 m radius	6	50
Dug wells	Latrine or septic tank soak-away within 10 m of the well	22	48
	Latrine/septic soak-away at higher ground than well, 10 to 30 m away.	30	65
	Other nearby sources of contamination such as wastewater drain, nearby rubbish dump, animal excreta, etc. within 10 m.	34	74
	Rope/bucket left at potentially contaminated point	22	48
	Height of apron wall less than 1 m and/or lack of top protection covering	29	63
	Depth and effectiveness of internal lining	28	61

**Table 2:** Bacteriological quality, depth, and sanitary risk levels of groundwater sources.

Source Type	Source ID	Latitude	Longitude	Depth (m)	*E. Coli* (cfu/100 mL)	Adenovirus	Rotavirus	Contaminant Risk Level
Boreholes	TG-8/50	5.88014	−0.099519	50	TNTC			Low
	TG-8/15	5.879999	−0.099432	15	TNTC			Low
	BH1	5.88046	−0.09626	10		+	-	Intermediate
	BH2	5.88288	−0.08747		485	~	-	High
	BH3	5.88172	−0.0877		425	-	-	High
	BH4	5.879241	−0.098909		825	~	~	Intermediate
	BH5	5.87838	−0.09472			~	-	Intermediate
	BH6	5.876314	−0.09294		187	~	I	Intermediate
	BH7	5.88815	−0.08936		TNTC	-	-	Very high
	BH8	5.88922	−0.08795		525	~	-	High
	BH9	5.88381	−0.07713		TNTC	-	-	High
	BH10	5.8859	−0.07521		113	I	-	High
	BH11	5.87991	−0.10614	30	135	~	-	Very high
	BH12	5.88671	−0.09589		88			Intermediate
Dug wells	DW1	5.8812	−0.1014	3.2	300	~	-	High
	DW2	5.877917	−0.10491	0.1	475	~	-	High
	DW3	5.88774	−0.08963	1.3	775	~	-	High
	DW4	5.88609	−0.09833	0.1	375	~	~	High
	DW5	5.88617	−0.09953	0.2	525	I	+	High
	DW6	5.878567	−0.094222	2.5	TNTC	~	-	Very high
	DW7	5.88239	−0.1064	1.4	TNTC	+	~	Very high
	DW8	5.880212	−0.104092	0.4	201	~	~	High
	DW9	5.88584	−0.09431	0.1	300	I	-	Very high
	DW10	5.88572	−0.08193	0.3	825	~	-	High
	DW11	5.88326	−0.09156	0.8	750	I	-	Very high

TNTC = too numerous to count, i.e., > 900 CFU/100 mL; + = positive (virus detected); - = negative (no virus detected); ~ = ambiguous (virus detected in one of the quadruplicate or quintuplicate samples, remaining samples were negative); I = inhibited PCR. Empty cell = not measured, or unknown depth.
